# Special Issue on Silicate Solid Waste Recycling

**DOI:** 10.3390/ma14143776

**Published:** 2021-07-06

**Authors:** Yue Xiao, Mujaheed Yunusa, Denis Jelagin, Hongbo Tan, Bohumír Strnadel

**Affiliations:** 1 State Key Laboratory of Silicate Materials for Architectures, Wuhan University of Technology (WUT), Wuhan 430070, China; mujaheedyunusa@whut.edu.cn (M.Y.); thbwhut@whut.edu.cn (H.T.); 2 Division of Building Materials, KTH Royal Institute of Technology, SE-100 44 Stockholm, Sweden; denis.jelagin@abe.kth.se; 3 Center of Advanced Innovation Technologies, VŠB-Technical University of Ostrava, 70833 Ostrava-Poruba, Czech Republic; bohumir.strnadel@vsb.cz

The reuse of industrial silicate solid wastes, including fly ash, slag, and waste rubber, is one of the most significant environmental and economic concerns worldwide. At present, many researchers are focusing on this topic, and construction and building materials have been considered as the best way to reuse such an enormous amount of industry silicate solid wastes [1]. For instance, steel slag has been successfully used in pavement engineering [2,3,4], fly ash is widely reused as cementitious materials in concrete [5,6,7], and waste tire rubber can be reused to improve the rutting resistance of asphalt pavement [8,9,10]. Along with these developments, there are still many challenges that should be further discussed.

This Special Issue, “Silicate Solid Waste Recycling”, aims to include the latest research findings on new materials and innovative technologies on solid waste recycling in construction and building materials. In total, it obtained 20 submissions, and 13 of them succeeded to the final publication, with valuable help from all the reviewers. Researchers from China, the United States, Russia, Czech Republic, and Malaysia chose to share their recent research findings via this particular issue.

Steel slag, a by-product from the steel-making industry treated as a typical solid waste, was reused in pavement engineering as aggregate. Feng used steel slag for pavement micro-surfacing and characterized its surface profile by laser scanning [3]. They found that steel slag can improve the skid resistance of pavement micro-surfacing due to its advantages of excellent polish resistance. Carbide slag was reused as an accelerator in calcium sulfoaluminate cement by Tan and Smirnova [11]. Research indicated that the setting time of cement could be significantly shortened due to the introduction of carbide slag, which can be widely used in special projects where a fast setting is required. Blast furnace slag, a by-product from iron production in blast furnaces, was granulated and reused to enhance the corrosion resistance of steel bar used in cement concrete [6]. The corrosion resistance of the cement-ground granulated blast furnace slag system and cement-fly ash system was compared by adding NaCl in the paste. While in the research from Choo, the coal fly ash, which is a combustion waste produced by a coal-fired power plant, was recycled in the production of clay ceramics [5]. An environment-friendly preparation method of reaction-bonded silicon carbide ceramics was reported by Ye [12].

Pavement engineering is one of the major application fields that can reuse massive amounts of industrial solid waste. This might be the reason that five papers out of thirteen focus on the recycling techniques in pavement engineering. Chang reported a bibliometric evaluation on the recent research activities on recycled construction materials in pavement engineering [13]. The research hotspots and development trends of pavement engineering were then forwarded by claiming that the rubber modified asphalt binder and reclaimed asphalt pavement (RAP) attracted the most attention. In this Special Issue, two papers focused on the crumb rubber modified asphalt binder [8,9] and another two papers focused on the performance enhancement of reclaimed asphalt pavement [14,15]. 

He reported the rheological properties of SBS/CRP (SBS, styrene-butadiene-styrene copolymer; CRP, crumb rubber powder) modified asphalt binder at different ageing levels [8]. Moreover, the ageing resistance of crumb rubber modified asphalt binder was discussed by Chen, by comparing microwave pre-treated crumb rubber with other traditional polymer modifiers [9]. Leng studied the effect of heating temperature on the compatibility of reclaimed asphalt mixture by employing a compaction energy ratio [14]. In comparison, Xiao tried to design a reclaimed asphalt mixture with extra high RAP content, close to 50% [15]. Basalt fiber was proposed to reinforce the performance of reclaimed asphalt mixture during their high rap recycling. 

The quality improvement method, including brick separation and surface treatment, on recycled aggregated from construction and demolition waste, was also included in this Special Issue [16]. Qiu shared their latest research on the asphalt–filler interaction analysis with the help of macro-rheological measurements [17]. Multiwall carbon nanotubes were used as a functional additive in the research from Smirnova to improve the electrical conductivity of cement matrices [18].

This editorial just briefly summarized the included papers. Please refer to the relevant papers for detailed information. The development of silicate solid waste recycling technologies is highly dependent on related scientific research. Durability is one of the major concerns during the material design with recycled solid waste. Life-cycle properties prediction and extension technics are urgently needed. The successful ending of this Special Issue is a new start for further silicate solid waste recycling. We hope that this Special Issue will support researchers to carry on further study in this area.
